# Fibroproliferative disorders and diabetes: Understanding the pathophysiologic relationship between Peyronie’s disease, Dupuytren disease and diabetes

**DOI:** 10.1002/edm2.195

**Published:** 2020-10-31

**Authors:** Martin K. Gelbard, Joel Rosenbloom

**Affiliations:** ^1^ Department of Urology David Geffen School of Medicine at UCLA Los Angeles CA USA; ^2^ Department of Dermatology and Cutaneous Biology The Joan and Joel Rosenbloom Research Center for Fibrotic Diseases Sidney Kimmel Medical College Thomas Jefferson University Philadelphia PA USA

**Keywords:** diabetes mellitus, Dupuytren disease, fibrosis, Peyronie's disease

## Abstract

**Introduction:**

Fibrosis is characterized by dysregulation and accumulation of extracellular matrix. Peyronie's disease and Dupuytren disease are fibroproliferative disorders of the tunica albuginea of the penis and fascia of the hand, respectively. Chronic hyperglycaemia due to diabetes mellitus can also lead to tissue injury and fibrosis. A meta‐analysis has shown a relationship between Dupuytren disease and diabetes (overall odds ratio, 3.1; 95% confidence interval, 2.7‐3.5). This review explores commonalities in the pathogenesis of Peyronie's disease, Dupuytren disease and diabetes.

**Methods:**

A search of the PubMed database was conducted using the search terms “diabetes” AND “Peyronie's disease”; and “diabetes” AND “Dupuytren.”

**Results:**

Genome‐wide association and gene expression studies conducted with tissue from people with Peyronie's disease or Dupuytren disease identified signalling pathways associated with wingless‐type mammary‐tumour virus integration site signalling, extracellular matrix modulation and inflammation. Biochemical studies confirmed the importance of these pathways in the pathogenesis of fibrosis with Peyronie's disease and Dupuytren disease. Dysregulation of matrix metalloproteinase activity associated with extracellular matrix breakdown was implicated in fibroproliferative complications of diabetes and in the aetiology of Peyronie's disease and Dupuytren disease. A notable percentage of people with diabetes have comorbid Peyronie's disease and/or Dupuytren disease.

**Conclusions:**

Studies have not been performed to identify fibroproliferative pathways that all 3 conditions might have in common, but data suggest that common pathways are involved in the fibroproliferative processes of Peyronie's disease, Dupuytren disease, and diabetes.

## Graphical abstract

Chronic hyperglycaemia associated with diabetes mellitus may result in the development of fibrosis, which can affect multiple organs. A notable percentage of people with diabetes have comorbid fibroproliferative disorders, specifically Dupuytren disease (fibrotic disorder of the fascia of the hand) or Peyronie's disease (fibrotic plaques in the tunica albuginea of the penis). Data from genome‐wide association and gene expression and biochemical studies suggest that the fibroproliferative processes of diabetes, Dupuytren disease and Peyronie's disease involve common pathophysiologic pathways.

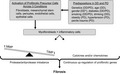

## 1 INTRODUCTION

In genetically susceptible individuals, chronic hyperglycaemia due to diabetes mellitus can cause tissue injury that may result in the development of fibrosis.^1^ Fibrosis is characterized by dysregulation, accumulation and change in the quality of the extracellular matrix resulting from an abnormal or uncontrolled tissue repair response. The extracellular matrix provides mechanical support for cells and facilitates cell‐cell communication through an insoluble network of collagen, elastins, structural glycoproteins, proteoglycans‐hyaluronans and integrins.^1^ The extracellular matrix is constantly being synthesized and degraded, and this turnover is important to ensure normal structure and function of organs and tissues. Fibrosis in people with diabetes can affect almost every organ in the body, including the heart, eyes, liver, kidney, skin and vascular system.^1^ Many people with diabetes have arthropathies, such as thickened skin and limited mobility of the joints of the hands and fingers, leading to flexion contracture(s).^2^ Endocrinologists may overlook limited joint mobility in people with diabetes or may not consider it a diabetes‐related complication.

Dupuytren disease is a progressive fibrotic disorder of the fascia of the palm and fingers. Mean age at onset is 49 years for individuals with a family history, compared with 55 years for those without a family history of Dupuytren disease.^3^ Nodules and cords fixed to the skin and palmar fascia disable normal movement of the hand and result in the pathology of Dupuytren contracture; a defect in wound repair is believed to initiate the fibrotic process of Dupuytren disease (in this review, ‘Dupuytren disease’ encompasses ‘Dupuytren contracture’).^4^, ^5^, ^6^ Several risk factors have been identified for Dupuytren disease, including diabetes, increasing age, being male, heavy alcohol consumption and smoking.^7^, ^8^ In people with diabetes and comorbid Dupuytren disease, the ring and middle fingers are typically affected, whereas in people with Dupuytren contracture but without diabetes, the ring and fifth (‘pinkie’) fingers typically are affected. A systematic review of 21 studies reported a relationship between Dupuytren disease and diabetes (overall odds ratio, 3.1; 95% confidence interval [CI], 2.7‐3.5; Table 1, Dupuytren disease).^9^ Furthermore, a strong association between both types of diabetes and Dupuytren disease was noted (Table 1).^9^ Investigators have identified a significant relationship between diagnosis of Dupuytren disease and increasing HbA_1c_ levels in 3418 people with comorbid Dupuytren disease and diabetes.^10^ A 2019 study reported a genome‐wide genetic correlation between Dupuytren disease and type 2 diabetes, although at a local genetic level, there was no correlation or pattern suggestive of a causal relationship between the 2 conditions.^11^

**Table 1:** Prevalence of people with diabetes and Dupuytren disease or Peyronie's disease

Dupuytren disease					
Meta‐analysis	Studies, n	Prevalence of DD in studies		OR (95% CI)*	*P* value*
		People with diabetes, mean % (range)	People without diabetes, mean % (range)		
Broekstra *et al* ^9^	21	31 (0.4‐69)	14 (0‐49)	Type 1 diabetes: 3.9 (2.5, 6.1) Type 2 diabetes: 3.0 (2.2, 4.2)	—

Peyronie's disease					
Study	Participants, n	Prevalence of diabetes in studies		OR (95% CI)*	*P* value*
		People with PD, %	People without PD, %		
Schwarzer *et al* ^17^	4432	18	6.0		*P* < .01^†^
Bjekic *et al* ^14^	82 with PD	32	11		*P* < .01^‡^
Mulhall *et al* ^18^	532	25	11	2.6 (1.3, 5.3)	*P* = .007^†^

Abbreviations: CI, confidence interval; DD, Dupuytren disease; OR, odds ratio; PD, Peyronie's disease.

* Age‐matched controls.

^†^ 2‐sided.

^‡^ Univariate logistic regression analysis.

Peyronie's disease, which is thought to have a pathophysiology generally similar to that of Dupuytren disease, is a progressive fibrotic disorder causing plaques in the tunica albuginea of the penis.^12^ The fibrotic plaques of Peyronie's disease can cause several abnormalities of the penis (eg curvature, shortening and narrowing), which can result in penile pain.^13^ Risk factors for Peyronie's disease include penile trauma, smoking, obesity and hypertension.^14^, ^15^, ^16^ Several studies have suggested a relationship between Peyronie's disease and diabetes (Table 1, Peyronie's disease).^14^, ^17^, ^18^ In addition, a 2019 Iranian study reported a prevalence of Peyronie's disease of 3.8% among 317 men with type 2 diabetes.^19^ In a retrospective study of 1622 men presenting to a urology health centre, the presence of type 2 diabetes (n = 387; 24%) was significantly associated with Peyronie's disease (*P* = .005); logistic regression analysis identified a significant association between glycated HbA_1c_> 69 mmol/mol (8.5%; eg poorly controlled) and Peyronie's disease (odds ratio [OR] = 1.6 [95% CI, 1.1‐2.5]; *P* = .025).^20^ Given that Peyronie's disease and Dupuytren disease appear to be localized manifestations of a systemic fibroproliferative pathologic process, the aim of this narrative review is to provide insights into commonalities in the genetics and pathogenesis of Peyronie's disease, Dupuytren disease and diabetes mellitus.

## 2 METHODS

A search of the PubMed database with no date restriction was conducted on 3 June 2019, using the following search terms: “diabetes” AND “Peyronie's disease”; and “diabetes” AND “Dupuytren.” Searches were limited to human studies and English‐language publications. Reference lists in all relevant publications were examined to identify additional articles for inclusion. An appendix has been developed (Appendix 1) to define genes and abbreviations discussed in the article. *P* values of ≤0.05 or <0.05 provided in the current article were statistically significant, per references, unless otherwise specified. Alternatively, use of ‘statistically significant’ was used, based on statistical methods used and interpretation by authors of each publication.

## 3 GENETIC LINKS FOR DEVELOPMENT OF PEYRONIE’S DISEASE AND DUPUYTREN DISEASE

Dupuytren disease most commonly affects men of northern European white descent.^21^ A study of a 5‐generation Swedish family suggested that Dupuytren disease is inherited as an autosomal dominant condition with incomplete penetrance.^22^ Two studies confirmed the heritability of Dupuytren disease, showing that siblings of people with Dupuytren contracture have an increased risk of developing the condition, with a 2.9‐fold greater (95% CI, 2.6‐3.3) risk^3^ to a 4.5‐fold greater (95% CI, 2.6‐7.8, *P* < .001) risk^23^ compared with the general population. A modelling study of 30,330 pairs of male Danish twins reported that genetic factors are important for the development of Dupuytren disease, with a heritability rate of 80% (95% CI, 69‐87).^24^ Nonshared environmental influences accounted for the remaining 20% of cases (95% CI, 13‐31).^24^ These results agree with earlier studies^3^, ^22^, ^23^ that indicated a strong genetic influence on Dupuytren disease development; however, no specific gene has been identified as its cause.^12^

A small US retrospective study of 67 people (mean age, 54 years; range, 17‐74 years) revealed that Peyronie's disease is less common in black individuals (13; 19%) than in northern European white individuals (52; 78%).^25^ A pedigree analysis of 3 families affected by both Peyronie's disease and Dupuytren disease (specifically, Dupuytren contracture) indicated that inheritance of Peyronie's disease is autosomal dominant with incomplete penetrance,^26^ similar to the way Dupuytren disease is inherited; however, specific genes that identify an increased risk of developing Peyronie's disease have not been identified.^12^ A study in the Netherlands noted that 22% of 415 men (mean ± SD age, 60 ± 12 years) who presented with Peyronie's disease had comorbid Dupuytren disease.^27^ A significant coexistence between Peyronie's disease and Dupuytren disease (specifically, Dupuytren contracture) was demonstrated in studies from the USA (n = 4/296 [1.4%]; *P* < .001),^28^ Brazil (n = 5/83 [6.0%]; *P* < .001)^29^ and Serbia/Montenegro (n = 32/82 [39%]; *P* < .01).^14^

## 4 GENETIC ASSOCIATIONS AND BIOCHEMICAL PATHWAYS LINKED TO THE AETIOLOGY OF FIBROSIS

As part of the normal wound‐healing process, myofibroblasts aid in wound closure through their contractile properties and secretion of growth factors and molecules that promote extracellular matrix formation.^30^ At the end of the wound‐healing process, myofibroblasts and vascular cells undergo apoptosis and are then removed from the wound site.^30^ Tissue and organ fibrosis is believed to result from the lack of myofibroblast apoptosis, which leads to extracellular matrix overproduction, tissue contraction and scar formation in conditions such as Dupuytren disease.^31^, ^32^

### 4.1 Genetics of Dupuytren disease and Peyronie's disease

Signalling pathways associated with the pathogenesis of fibrosis in Peyronie's disease and Dupuytren disease include wingless‐type mammary‐tumour virus integration site (WNT) signalling, extracellular matrix modulation and inflammation.^33^, ^34^, ^35^, ^36^, ^37^

#### 4.1.1 Studies in Dupuytren disease

Dupuytren disease progression involves 3 stages: stage 1, proliferation of fibroblasts; stage 2, differentiation of fibroblasts into myofibroblasts; and stage 3, formation of mature type 1 collagen.^38^, ^39^, ^40^ The WNT gene family comprises several structurally related genes that encode glycoproteins and extracellular signalling molecules.^33^ Immunohistochemical analysis of palmar fascia and cords from 20 people with Dupuytren disease showed that WNT genes modulate the proliferation and differentiation of fibroblasts in Dupuytren disease.^41^ A genome‐wide association study (GWAS), followed by a meta‐analysis of 2325 people with Dupuytren disease and 11,562 controls,^33^ identified 11 single‐nucleotide polymorphisms (SNPs) from 9 different loci (Table 2).^33^, ^34^, ^35^, ^36^, ^42^, ^43^ A total of 6 loci were statistically significantly associated with the WNT signalling pathway: *WNT4*, secreted frizzle‐related protein *(SFRP4*), *WNT2*, R‐spondin (*RSPO2*), heparan sulphate 6‐O‐endosulfatase (*SULF1*), and *WNT7B*.^33^ On the basis of the GWAS results and meta‐analysis, the investigators hypothesized that modulation of WNT signalling could increase the proliferation of fibroblasts in the fascia of the hand and result in nodule formation.^33^

**Table 2:** Genetic links to signalling pathways implicated in the fibroproliferative processes of Dupuytren disease and Peyronie's disease^33^, ^34^, ^35^, ^36^, ^42^, ^43^

Pathway	Dupuytren disease	Peyronie's disease
WNT signalling^33^, ^34^, ^35^, ^36^, ^42^		
*EPDR1*	✓	—
*RSPO2*	✓	—
*SFRP4*	✓	—
*SULF1*	✓	—
*WNT2*	✓	✓
*WNT4*	✓	—
*WNT7B*	✓	—
ECM modulation^34^, ^35^, ^43^		
*ACAN*	✓	*—*
*ADAMTS‐14*	✓	*—*
*CHST6*	✓	*—*
*DDR2*	✓	*—*
*ITGA11*	✓	*—*
*MMP‐1*	✓	*—*
*MMP‐2*	✓	✓
*MMP‐9*	✓	✓
*MMP‐13*	✓	—
*MMP‐14*	✓	—
*OSF‐1*	✓	✓
*OSF‐2*	✓	✓
*Rho‐GDP1*	✓	✓
*TIMP‐1*	✓	—
*TMSβ‐4*	✓	✓
*TMSβ‐10*	✓	✓
Inflammation^34^, ^35^		
*GCKR/MAP4K5*	✓	*—*
*DCN (also known as PGS2)*	✓	✓

Abbreviations: *ACAN*, aggrecan; *ADAMTS*, a disintegrin and metalloproteinase domain with thrombospondin motif; *CHST*, carbohydrate sulfotransferase; *DCN*, decorin; *DDR*, discoidin domain receptor; ECM, extracellular matrix; *EPDR*, ependymin‐related protein*; GCKR/MAP4K*, germinal center kinase‐related/mitogen‐activated protein kinase kinase kinase kinase; *ITGA*, integrin subunit α; MMP, matrix metalloproteinase; *OSF*, osteoblast‐specific factor; *PGS*, bone proteoglycan; *RHOGDP*, RhoGDP dissociation inhibitor; *RSPO*, R‐spondin; *SFRP*, secreted frizzle‐related protein; *SULF*, sulfatase; *TIMP*, tissue inhibitors of metalloproteinases; *TMSβ*, thymosin *β*; WNT, wingless‐type mammary‐tumour virus integration site.

A larger GWAS of people with Dupuytren disease (n = 3871) compared with controls (n = 4686) identified statistically significant associations for 14 variants.^34^ Forty‐five statistically significant SNPs were replicated in people with Dupuytren contracture (n = 4041) and corresponding controls (n = 8251) and identified genome‐wide significant associations for 15 new loci, in addition to confirming the 9 previously reported loci.^33^, ^34^ Five previously identified loci associated with the WNT signalling pathway (*WNT2*, *WNT4*, *WNT7B*, *RSPO2* and *SFRP4*) were confirmed in this study.^34^ The most significantly associated SNP identified by direct genotyping was located in the intron of ependymin‐related protein 1 (*EPDR1)* and upstream of *SFRP4*.^34^ *EPDR1* encodes a type II transmembrane protein, and *SFRP4* encodes a secreted protein homologous to the membrane‐bound WNT Frizzled receptors.^34^ The authors hypothesized, from functional studies in primary myofibroblasts derived from surgically resected Dupuytren disease tissue, that decreased secretion of Sfrp4 may increase Wnt3a signalling via the noncanonical pathway, and the authors concluded that changes in WNT signalling play a role in the fibrotic phenotype (Table 3).^32^, ^34^, ^60^

**Table 3:** Proteins linked to signalling pathways implicated in the fibroproliferative processes of Dupuytren disease, Peyronie's disease, and diabetes mellitus^32^, ^34^, ^60^

Pathway	Dupuytren disease	Peyronie's disease	Diabetes
WNT signalling^34^, ^42^, ^44^, ^45^, ^46^, ^47^			
*β‐catenin*	✓	✓	—
*Dkk‐1*	✓	—	✓
*Epdr1*	✓	—	—
*Sclerostin*	—	—	✓
*Sfrp4*	✓	—	—
*Wnt2*	✓	—	—
*Wnt3A*	✓	—	—
*Wnt7b*	✓	—	—
ECM modulation^37^, ^56^			
*MMP/TIMP*	✓	✓	✓
*MMP‐2*	✓	—	✓
*MMP‐9*	—	—	✓
*MMP‐14*	✓	—	—
*TIMP‐1*	✓	—	—
*TGF‐β*	—	—	✓
*ADAMTS‐2*	✓	—	—
*ADAMTS‐3*	✓	—	—
*ADAMTS‐12*	✓	—	—
*ADAMTS‐16*	✓	—	—
Inflammation^32^, ^37^, ^45^, ^54^, ^55^, ^57^, ^58^, ^59^, ^60^			
*TGF‐β*	✓	✓	✓
*TNF*	✓	—	—
*Interleukin‐6*	✓	—	—

Abbreviations: ADAMTS, a disintegrin and metalloproteinase domain with thrombospondin motif; Dkk, Dickkopf; ECM, extracellular matrix; Epdr, ependymin‐related protein; MMP, matrix metalloproteinase; Sfrp, secreted frizzle‐related protein; TGF‐β, transforming growth factor beta; TIMP, tissue inhibitors of metalloproteinases; TNF, tumour necrosis factor; WNT/Wnt, wingless‐type mammary‐tumour virus integration site [protein/gene].

The GWAS also identified SNPs in genes involved in extracellular matrix modulation, including discoidin domain receptor 2 (*DDR2*), matrix metalloproteinase 14 (*MMP‐14*), integrin α11, aggrecan (*ACAN*) and carbohydrate sulfotransferase 6 (*CHST6*).^34^ In addition, the GWAS results suggested that an SNP associated with a gene, germinal center kinase‐related (*GCKR*), may be involved in cross‐talk between inflammatory mediators (eg tumour necrosis factor [TNF]) and WNT signalling.^34^ Another GWAS and meta‐analysis study, of 1580 people with Dupuytren disease and 4480 controls, confirmed that the genetic basis for Dupuytren disease involves genes from the WNT signalling pathway.^61^

Whole transcriptome data from tissue derived from 12 people with Dupuytren disease and 12 controls identified the WNT/β‐catenin pathway as being significantly dysregulated in Dupuytren disease.^61^ Similarly, involvement of WNT signalling due to high levels of β‐catenin was suggested from studies performed with Dupuytren disease clinical lesions,^44^ which biochemically confirmed the GWAS results.^33^, ^34^, ^61^ Furthermore, WNT pathway loci identified from a GWAS were evaluated in a subsequent differential expression and immunohistochemical study performed with Dupuytren disease tissues (nodules and cords) and unaffected transverse palmar fascia (control tissues) from the same 8 individuals.^42^ The investigators found that WNT pathway‐related genes were differentially regulated in tissue samples of Dupuytren disease as compared with control tissues. In nodules, *WNT2* was downregulated (ninefold, *P* < .01) and *WNT7b* was upregulated (fivefold, *P* < .01), while immunohistochemical staining showed significant downregulation of Wnt2 protein in cords (*P* < .05) and upregulation of Wnt7b protein in nodules (*P* < .05).^42^ *SFRP4* was upregulated twofold in nodules and cords (*P* < .01), but no significant differences were seen by immunohistochemical staining.^42^ No significant differences were observed by mRNA expression or immunohistochemical staining for *WNT4*, *RSPO2* or *SULF1*.^42^ Immunohistochemical staining of β‐catenin was significantly higher in Dupuytren disease nodules, specifically in the nucleus (*P* < .05), compared with normal tissues.^42^ The investigators postulated that the co‐localization of β‐catenin and Wnt7b may suggest Wnt7b is the protein causing activation of the WNT pathway in Dupuytren disease.^42^

An immunohistochemical study with nodule and cord tissue from 40 people with Dupuytren disease reported a correlation between the number of macrophages and number of myofibroblasts in nodules (*r*_s_ = 0.58, *P* < .05).^4^ It has also been shown that mesenchymal stem cells, which are precursors for myofibroblast differentiation, may be present in fat and dermal tissues surrounding Dupuytren nodules.^47^ Furthermore, flow cytometry data indicated that immune cells (primarily macrophages) from disaggregated Dupuytren nodule tissue secrete a variety of proinflammatory cytokines (eg transforming growth factor beta [TGF‐β1], TNF and interleukin‐6).^32^ This study also compared palmar fibroblasts from people with Dupuytren disease to nonpalmar fibroblasts from these same individuals or to palmar cells from healthy individuals to evaluate the effects of cytokine activity on contraction and profibrotic signalling pathways. Exogenous addition of recombinant human TNF increased contraction of fibroblasts from people with Dupuytren disease but not in control tissues,^32^ and isolated cells from Dupuytren nodules secreted TNF at concentrations (mean ± SD, 78 ± 26 pg/mL) necessary for differentiation of palmar dermal fibroblasts into myofibroblasts.^32^ Consistent with GWAS data,^33^, ^34^, ^61^ biochemical studies confirmed that myofibroblast differentiation occurred via the WNT signalling pathway.^32^

TGF‐β1 stimulates myofibroblast differentiation and is a primary inducer of fibrosis in multiple tissues, such as breast,^62^ kidney,^63^ heart,^64^ lung^65^ and liver.^66^ In dermal fibroblasts obtained from 12 people with Dupuytren disease (mean age, 55 years; range, 42‐72 years), the production of TGF‐β1 was greater than that of TGF‐β2, and addition of TGF‐β1 (5 ng/mL) to Dupuytren disease cultures increased mitogenesis by up to fivefold.^57^ TGF‐β1 also plays a role in Peyronie's disease pathogenesis. Peyronie's disease plaques arise following penile trauma leading to deposition of fibrin, which attracts inflammatory cells (eg macrophages, mast cells), which in turn secrete TGF‐β1.^58^, ^59^, ^60^

#### 4.1.2 Studies in both Dupuytren disease and Peyronie's disease

A comparative gene expression study of tissues (n = 9 per group) from Peyronie's disease plaques, Dupuytren disease palmar fascia nodules and corresponding healthy tunica albuginea identified a common pathophysiology with gene families that involve collagen degradation and myofibroblast differentiation.^35^ Genes that were upregulated (mean fold change ± SE) in both Dupuytren disease nodules and Peyronie's disease plaques compared with healthy tissue included MMPs (*MMP‐2*: Dupuytren disease, 29.0 ± 10.0 [n = 9]; Peyronie's disease, 4.7 ± 2.6 [n = 2]; *MMP‐9*: Peyronie's disease, 50.8 ± 0.8 [n = 2]) and peptide activators of MMPs, thymosins (*TMSβ‐10*: Dupuytren disease, 5.9 ± 2.6 [n = 9]; Peyronie's disease, 5.5 ± 1.3 [n = 5]; *TMSβ‐4*: Dupuytren disease, 5.9 ± 1.5 [n = 8]; and Peyronie's disease, 2.5 ± 0.9 [n = 5]).^35^ Thymosin genes also were upregulated in Peyronie's disease fibroblasts (n = 2; *TMSβ‐10*, 2.0 ± 0.26; *TMSβ‐4*, 1.9 ± 0.1).^35^ A subsequent gene expression study, which did not apply correction for multiple testing, compared tissue from people with Dupuytren disease (n = 20; age range, 42‐83 years) versus normal palmar fascia (n = 20; age range, 25‐84 years). This study confirmed significant upregulation of *MMP‐2* (*P* < .001) and identified several additional genes that were significantly upregulated in Dupuytren disease nodule tissue, including *MMP‐1* (*P* < .001), *MMP‐13* (*P* < .001) and *MMP‐14* (*P* < .001); 3 members of the ADAMTS (a disintegrin and metalloproteinase domain with thrombospondin motif) family, particularly *ADAMTS‐14* (*P* < .001); and tissue inhibitor of matrix metalloproteinase 1 (*TIMP‐1*, *P* < .001).^43^

The individuals from that gene expression study were monitored for a mean duration of 14 months, during which clinical parameters (eg range of motion, grip strength) were scored in the preoperative, early postoperative (3 months) and final postoperative periods for the affected digit.^56^ Analysis of correlations (R value) between clinical outcomes and gene expression showed significant results for preoperative grip strength (n = 7; *MMP‐2*, 0.86 [*P* = .014]; *MMP‐14*, 0.93 [*P* = .003]; *TIMP‐1*, −0.93 [*P* = .003]; *ADAMTS*‐*2*, 0.79 [*P* = .036]; *ADAMTS‐3*, 0.79 [*P* = .036]; *ADAMTS‐12*, 0.89 [*P* = .007]; and *ADAMTS‐16*, 0.86 [*P* = .014]) and total further flexion (n = 17; *MMP‐2*, 0.68 [*P* = .002]; *MMP‐14*, 0.58 [*P* = .014]; *ADAMTS‐2*, 0.65 [*P* = .005]; *ADAMTS‐3*, 0.50 [*P* = .039]; *ADAMTS‐12*, 0.52 [*P* = .033]; and *ADAMTS‐16*, 0.49 [*P* = .047]).^56^ The importance of MMPs in the aetiology of Dupuytren disease was first described in a case series in which 3 out of 12 people with gastric cancer treated with a nonspecific MMP inhibitor developed a condition resembling Dupuytren disease.^67^ The investigators hypothesized that the pathogenesis of Dupuytren disease may involve a reduction in the MMP:TIMP ratio, leading to increased formation of collagen and connective tissue.^67^

A study of tunica albuginea plaque fibroblasts from 36 people (mean age, 56 years) with stable Peyronie's disease (mean, 2.3 years) confirmed the importance of the MMP and TGF‐β pathways in the fibrotic process of Peyronie's disease via use of protein microarrays and Western immunoblotting.^37^ In a comparative gene expression study that included Peyronie's disease plaque, Dupuytren disease nodules and healthy tissues, the distribution (mean fold change ± SE) of osteoblast‐specific factors (OSFs; genes involved in ossification) in both Dupuytren disease nodules and Peyronie's disease plaques was as follows: *OSF‐1*: Dupuytren disease, 5.6 ± 1.4 (n = 5); Peyronie's disease, 4.3 ± 0.5 (n = 3); and *OSF‐2*, Dupuytren disease, 26.7 ± 12.7 (n = 4).^35^ *RHOGDP* dissociation inhibitor 1 (Rho‐GDI1; a gene involved in myofibroblast differentiation) was also detected in Dupuytren disease nodules (3.5 ± 1.4 [(n = 6]) and Peyronie's disease plaques (18.3 ± 2.4 [n = 2]).^35^ Bone proteoglycan II precursor (*PGS2*, decorin), an inhibitor of TGF‐β1 with a role in fibroblast growth and collagen synthesis, was upregulated only in Peyronie's disease (Dupuytren disease, 0.5 ± 0.1 [n = 6]; Peyronie's disease, 2.5 ± 0.3 [n = 5]).^35^

To determine whether the loci of Dupuytren disease‐associated variants were involved in susceptibility to Peyronie's disease, SNPs in 9 genes (*WNT4*, *SFRP4*, *WNT2*, *SULF1*, *RSPO2*, doublesex and mab‐3 related transcription factor 2 *[DMRT2]*, zinc finger protein 264 *[ZNF264]*, MAF bZIP transcription factor B *[MAFB]* and *WNT7B*) previously identified in a GWAS^33^ were genotyped in 111 men with Peyronie's disease and 490 male controls.^36^ A significant association was observed with *WNT2* (*P* = .002; after Bonferroni correction), which is believed to protect against development of Peyronie's disease.^36^ Based on power calculations and results when excluding participants with comorbid Dupuytren disease, the investigators suggested that *WNT2* is more strongly associated with susceptibility to Peyronie's disease than to Dupuytren disease.^36^ Proteins associated with wound healing and fibrosis were significantly elevated in primary cell cultures of Peyronie's disease plaque tissue (n = 11) compared with healthy tunica albuginea (n = 11), including smooth muscle α‐actin (*P* < .01) and β‐catenin (*P* < .05), as confirmed by Western immunoblotting.^45^ Increased staining for TGF‐β1 was observed in cells from people with Peyronie's disease compared with those from controls.^45^ These data are consistent with the results from gene expression and biochemical studies that have identified processes associated with wound healing and tissue fibrosis in Peyronie's disease pathophysiology.

### 4.2 Genetics of diabetes

TGF‐β has been shown to promote renal cell hypertrophy and extracellular matrix accumulation in diabetes,^54^, ^55^ which is generally similar to the aetiologic processes of Dupuytren disease^57^ and Peyronie's disease.^35^, ^37^ In a hyperglycaemic clamp study, increases in blood glucose levels in 13 healthy volunteers (mean age, 39 years) caused a sevenfold increase in urinary excretion of TGF‐β1 (*P* = .002) compared with baseline,^68^ and data suggest that increased blood glucose levels can activate TGF‐β.^69^ Decorin, an inhibitor of TGF‐β1 that is upregulated in people with Peyronie's disease,^35^ was also upregulated by 650% ± 60% of control values in the kidney tissue of people with diabetic nephropathy compared with healthy kidney tissue.^55^ In advanced stages of diabetic nephropathy, decorin deposition was found in fibrotic areas and colocalized with type I collagen.^55^

**FIGURE 1:**
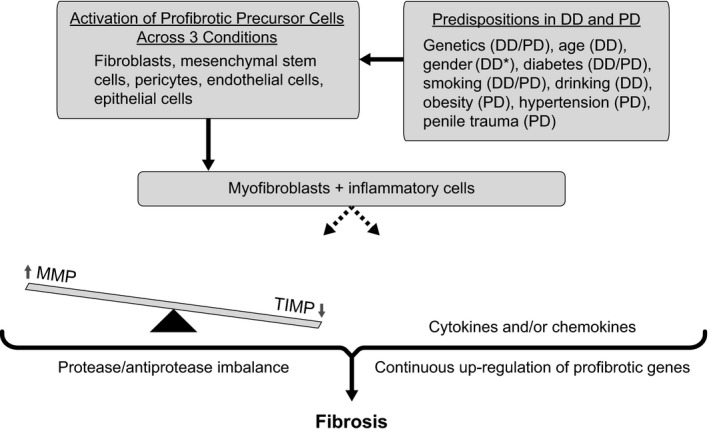
Proteins implicated in the fibroproliferative processes of Dupuytren disease, Peyronie's disease, and diabetes mellitus.^1^, ^12^, ^21^, ^35^, ^37^, ^47^, ^48^, ^52^, ^56^, ^70^, ^71^ *Peyronie's disease occurs in males only. DD, Dupuytren disease; MMP, matrix metalloproteinase; PD, Peyronie's disease; TIMP, tissue inhibitors of MMPs

Dysregulation of MMP activity associated with breakdown of constituents of the extracellular matrix (Figure 1)^1^, ^12^, ^21^, ^35^, ^37^, ^47^, ^48^, ^52^, ^56^, ^70^, ^71^ has been implicated in type 2 diabetes and in fibroproliferative complications associated with diabetes,^48^ and it also has been implicated in the aetiology of Dupuytren disease^56^ and Peyronie's disease.^37^ When quantified using enzyme‐linked immunosorbent assay (ELISA; median ng/mL [interquartile range]), MMPs were significantly elevated in 181 people with type 2 diabetes compared with 165 controls (MMP‐2: diabetes group, 1363.4 [1250.3‐1461.3]; control group, 639.3 [415.0‐804.0]; MMP‐9: diabetes group, 523.4 [476.5‐566.6]; control group, 55.3 [39.2‐68.2]; both, *P* < .001).^48^

Another complication of diabetes, diabetic retinopathy, is characterized by proliferation of fibrovascular tissue from the degradation of extracellular matrix components, a main cause of visual impairment.^49^ Concentrations of vitreous MMP‐2 and MMP‐9, as measured by ELISA, were significantly increased in 24 people with diabetic retinopathy (*P* < .05) compared with controls.^49^ A subsequent study in participants with type 1 diabetes (n = 47; age ± SD, 40 ± 14 years; range, 20‐65 years) reported that MMP‐9 was significantly increased in the systemic circulation compared with controls (n = 35; age ± SD, 41 ± 9 years; range, 24‐56 years; *P* < .001),^53^ while nonsignificant increases in serum TIMP‐1 were observed in participants with type 1 diabetes versus controls (*P* = .07).^53^ Significantly higher levels of MMP‐9 also were seen when comparing participants with type 1 diabetes with, versus without, retinopathy (*P* < .05).^53^

Evidence supports the theory that hyperglycaemia associated with diabetes causes dysregulation of MMPs in primary cells from the vasculature (macrophages and endothelial cells), and that both MMP‐2 and MMP‐9 may be involved in the rupture of atherosclerotic plaques.^69^, ^72^ Increased circulating levels of MMP‐2 were associated with microangiopathy in 25 children and adolescents with type 1 diabetes (median age, 11 years; range, 8‐12 years) compared with 19 controls (median age, 12 years; range, 11‐13 years).^50^ At baseline, MMP‐2 levels and activity were significantly higher in people with type 1 diabetes with or without complications compared with the control group of people without diabetes (*P* < .001 for both).^50^ Participants who experienced complications due to microangiopathy during follow‐up had significantly higher MMP‐2 levels (*P* = .009) and enzymatic activity (*P* < .001) compared with participants without complications.^50^ The significant differences seen in MMP‐2 levels and activity between participants with type 1 diabetes and microangiopathic complications versus controls persisted for 5 years (*P* < .001 for both).^50^

Systemic concentrations of MMP‐2 and MMP‐9 are increased in people with type 2 diabetes and peripheral arterial disease.^51^ In a study of people with type 2 diabetes with (n = 51) or without (n = 42) peripheral arterial disease and in healthy volunteers (n = 23), plasma MMP‐2 and MMP‐9 levels (mean ± SD, ng/mL) quantified using ELISA were significantly increased in the diabetes group with peripheral arterial disease compared with healthy volunteers (MMP‐2: peripheral arterial disease, 1121 ± 456; controls, 701 ± 362; *P* < .01; MMP‐9: peripheral arterial disease, 62 ± 30; controls, 25 ± 17; *P* < .001).^51^ The plasma levels of MMP‐2 were not significantly different between participants with type 2 diabetes without peripheral arterial disease or healthy volunteers; however, there was a significant difference in MMP‐9 levels between participants with type 2 diabetes with peripheral arterial disease and without peripheral arterial disease (with peripheral arterial disease, 62 ± 30; without peripheral arterial disease, 39 ± 24; *P* < .01).^51^

Mean (± standard error of the mean [SEM]) urine and plasma concentrations of MMP‐2 were significantly elevated in people with type 1 diabetes (n = 93) compared with age‐matched healthy volunteers (n = 50) (urine, pg/mL: diabetes group, 184.9 ± 31.3; control group, 48.4 ± 10.2; *P* < .001; plasma, mg/mL: diabetes group, 274.0 ± 20.0; control group, 191.2 ± 14.5; *P* < .005).^52^ Urine MMP‐2 results were similar whether analysed as MMP‐2‐to‐creatinine ratio or total MMP‐2 excreted per day.^52^ The amount of active MMP‐2 (ng/mL, mean ± SEM) was also significantly elevated in people with type 1 diabetes compared with age‐matched healthy volunteers (diabetes group, 292.7 ± 190.2; control group, 193.3 ± 163.0; *P* < .005).^52^ MMP‐2 concentrations in urine correlated with clinical parameters associated with increased risk for diabetic nephropathy (eg elevation in glycated HbA_1c_, prolonged duration of diabetes, renal hyperfiltration and microalbuminuria).^52^ Thus, evidence supports a role for MMPs in the pathogenesis of diabetes complications of nephropathy,^52^ retinopathy,^49^, ^53^ peripheral arterial disease^51^ and microangiopathy.^50^

WNT signalling pathways have been implicated in fibroproliferative complications of diabetes such as increased carotid intima‐media thickness, a noninvasive indicator of subclinical atherosclerosis.^46^ For example, serum levels of inhibitors of the WNT β‐catenin pathway were significantly higher in 40 women with type 2 diabetes (mean age ± SD, 64 ± 8 years) than in 40 healthy volunteers (mean age, 62 ± 8 years) when measured by ELISA (mean ± SD; pmol/L) (sclerostin: diabetes, 53.2 ± 10.9; control group, 47.5 ± 12.6; *P* < .05; Dickkopf‐1 gene (Dkk‐1): diabetes, 12.9 ± 10.3; control group, 9.1 ± 5.7; *P* < .05).^46^ A significant difference in maximal carotid intima‐media thickness (mean ± SD) was seen in the group with diabetes (0.9 ± 0.2 mm) compared with the control group (0.8 ± 0.1 mm; *P* < .05).^46^ After adjusting for age, there was a significant negative univariate correlation between serum sclerostin and carotid intima‐media thickness in the diabetes group (*r* = ‒0.42; *P* = .006) and between serum Dkk‐1 and carotid intima‐media thickness in the diabetes group (*r* = ‒0.48; *P* = .001); no significant correlations were seen between these serum markers and carotid intima‐media thickness in the control group.^46^ The investigators hypothesized that increased levels of these WNT pathway inhibitors could disrupt WNT pathway signalling, which is activated during atherosclerosis.^46^

## 5 TREATMENT OF DUPUYTREN DISEASE AND PEYRONIE’S DISEASE

### 5.1 Dupuytren disease

There is a lack of strong clinical evidence for the use of nonoperative treatments for early‐stage Dupuytren disease (eg physiotherapy, splinting and local radiotherapy).^73^ For patients with established flexion deformities, surgery is considered when hand function is affected and the digits are flexed ≥ 15°.^74^ A popular surgical technique for advanced Dupuytren disease (ie Dupuytren contracture) is removal of the diseased tissue or cords (fasciectomy).^75^ A minimally invasive surgical technique, percutaneous needle fasciotomy, mechanically divides the cords to straighten the affected finger.^76^ In one study, assessment of 292 treated joints 5 years postprocedure showed a recurrence rate for Dupuytren disease, defined as ≥ 20° of worsening, in the metacarpophalangeal joint of 22% for percutaneous needle fasciotomy and 5.3% for limited fasciectomy.^77^ The high rate of recurrence after surgical procedures implies that surrounding tissue may be involved in the disease aetiology.^47^ Excision of the skin over the nodule and perinodular fat, or dermofasciectomy, has a reduced rate of recurrence, supporting the hypothesis that surrounding tissue may be involved in the fibroproliferative process of Dupuytren disease.^47^

Approved by the US Food and Drug Administration (FDA) in 2010, injectable collagenase clostridium histolyticum (CCH) is a minimally invasive treatment for adults with Dupuytren contracture with a palpable cord, and the administration guidelines allow concurrent treatment of up to two affected joints in the same hand.^78^ Support for FDA approval included data from two phase 3, randomized, placebo‐controlled studies, in which clinical success (ie correction of Dupuytren contracture to ≤ 5°) was significantly higher with CCH versus placebo when measured as percentage of primary joints with reduction in contracture at 30 days after the final injection (64% vs 6.8%; *P* < .001 for one study; and 44% vs 4.8%; *P* < .001 for the second study).^79^, ^80^ The 5‐year recurrence rate, defined as ≥ 20° of worsening posttreatment with CCH, was 47% of 623 successfully treated joints in another study.^81^

One drawback to current treatments for Dupuytren disease is that they are employed in more advanced disease settings.^73^ A systematic review highlighted the lack of evidence for treatments that target the early stages of Dupuytren disease fibroproliferative processes.^73^ TNF has been implicated in the differentiation of fibroblasts into myofibroblasts in people with Dupuytren disease, and an in vitro study showed that anti‐TNF neutralizing antibody reduced isometric contraction of myofibroblasts from participants with Dupuytren disease in a dose‐dependent manner.^32^ A dose‐escalation study with the anti‐TNF antibody adalimumab (15 mg, 35 mg, or 40 mg) or placebo (saline) was performed in 28 participants with early‐stage Dupuytren disease.^82^ Two weeks after administration of the antibody, Dupuytren nodules were excised and protein expression (mean ± SD) was analysed; levels of α‐smooth muscle actin, a biomarker of myofibroblast differentiation, were significantly reduced in treated participants compared with placebo (40‐mg treatment group, 1.1 ± 0.1 ng/µg of total protein; placebo group, 1.5 ± 0.1 ng/µg of total protein; *P* = .006).^82^ Procollagen type 1 was also significantly reduced compared with placebo (40‐mg treatment group, 474 ± 84 pg/µg of total protein; placebo group, 817 ± 78 pg/µg of total protein; *P* = .019).^82^ The results indicate that treatment with an anti‐TNF antibody downregulates the myofibroblast phenotype and suggest that treatments targeting the WNT signalling pathway may be promising for the treatment of early‐stage Dupuytren disease.^82^

### 5.2 Peyronie's disease

Treatment options for Peyronie's disease include oral therapies (eg pentoxifylline), traction, injection therapy (interferon‐α2b, calcium channel blockers) and surgery; however, many of these treatment options have not yet been evaluated in randomized, controlled clinical trials.^83^ In addition to Dupuytren contracture, CCH is approved by the FDA for the treatment of adult men with Peyronie's disease who have a palpable plaque and penile curvature of ≥ 30° at the start of therapy.^78^ Collagenase clostridium histolyticum is the only medication currently approved for Peyronie's disease, and the clinical safety and efficacy of CCH were shown in two large, 12‐month, double‐blind, placebo‐controlled clinical trials (Investigation for Maximal Peyronie's Reduction Efficacy and Safety Studies [IMPRESS] I & II^77^) and a 9‐month, open‐label, follow‐up trial in men who had received placebo during IMPRESS I and II.^84^, ^85^

## 6 CONCLUSION

Believed to occur as part of a systemic fibroproliferative disorder, Peyronie's disease and advanced Dupuytren disease (ie Dupuytren contracture) share some common traits, including inflammation, collagen degradation, ossification and myofibroblast differentiation. Inheritance of Dupuytren disease or Peyronie's disease is autosomal dominant with incomplete penetrance; however, specific genes that identify an increased risk of developing Peyronie's disease or Dupuytren disease have not been identified. Even though a notable percentage of people with diabetes have comorbid Peyronie's disease and/or Dupuytren disease, studies have not been performed to identify fibroproliferative pathways that all 3 conditions might have in common. Based on this review of the literature, data suggest a possible common link among the pathways involved in the fibroproliferative processes of Peyronie's disease, Dupuytren disease and diabetes.

## CONFLICT OF INTEREST

Martin K. Gelbard reports serving as a speaker for Endo Pharmaceuticals Inc and being a consultant for BioSpecifics Technologies Corp. Joel Rosenbloom reports having served as a consultant to Endo Pharmaceuticals Inc

## AUTHOR CONTRIBUTIONS

Martin K. Gelbard and Joel Rosenbloom contributed to the conception of the manuscript, critically reviewed and revised drafts of the manuscript during development and approved the final version of the manuscript submitted for publication; both authors are accountable for all aspects of the scientific information presented in the manuscript.

## Data Availability

Data sharing is not applicable to this article as no new data were created or analysed in this study.

## APPENDIX 1

### GENE/PROTEIN NAMES AND ACTIVITY

ACAN, aggrecan (extracellular matrix modulation)

ADAMTS, a disintegrin and metalloproteinase domain with thrombospondin motif (tissue development and maintenance)

CHST6, carbohydrate sulfotransferase 6 (extracellular matrix modulation)

DCN, decorin (an inhibitor of TGF‐β and a part of fibroblast replication/collagen synthesis)

DDR2, discoidin domain receptor 2 (extracellular matrix modulation)

Dkk‐1, Dickkopf‐1 gene (antagonist for signalling [inhibits β‐catenin signalling])

DMRT2, doublesex and mab‐3 related transcription factor 2

EPDR1, ependymin‐related protein 1 (myofibroblast contractility)

GCKR/MAP4K5, germinal center kinase‐related/mitogen‐activated protein kinase kinase kinase kinase 5 (cross‐talk between inflammatory mediators)

ITGA11, integrin subunit alpha 11 (extracellular matrix modulation)

MAFB, MAF bZIP transcription factor B (tissue development and cellular differentiation)

MMP, matrix metalloproteinase (extracellular matrix modulation [collagen degradation])

OSF, osteoblast‐specific factor (osteoblast recruitment for ossification)

RHOGDP1, RhoGDP dissociation inhibitor 1 (myofibroblast differentiation)

PGS2, bone proteoglycan (preferred term is decorin; an inhibitor of TGF‐β and a part of fibroblast replication/collagen synthesis)

RSPO2, R‐spondin (agonist of WNT signalling)

SFRP4, secreted frizzle‐related protein (antagonist of WNT signalling)

SULF1, heparan sulphate 6‐O‐endosulfatase (agonist of WNT signalling)

TIMP‐1, tissue inhibitors of metalloproteinases 1

TNF, tumour necrosis factor (proinflammatory cytokine)

TGF‐β, transforming growth factor beta (inflammatory cytokine; promotes fibrogenesis)

TMSβ, thymosin beta (MMP activator)

WNT, wingless‐type mammary‐tumour virus integration site (extracellular matrix modulation)

ZNF264, zinc finger protein 264 (transcription factor found in skeletal muscle)
